# Anatomic measurement of osseous parameters of the glenoid

**DOI:** 10.1038/s41598-022-17783-y

**Published:** 2022-08-04

**Authors:** Jing Zhou, Bin Zhong, Rongmei Qu, Lei Qian, Zeyu Li, Chang Liu, Zhaoming Xiao, Guangwei Xu, Haibin Liang, Kuanhai Wei, Jun Ouyang, Jingxing Dai

**Affiliations:** 1 Department of Anatomy, Youjiang Medical University for Nationalities, Baise, 533000 China; 2 Guangdong Provincial Key Laboratory of Medical Biomechanics and Guangdong Engineering Research Center for Translation of Medical 3D Printing Application and National Virtual and Reality Experimental Education Center for Medical Morphology (Southern Medical University) and National Key Discipline of Human Anatomy, School of Basic Medical Sciences, Southern Medical University, 1023 Shatai South Road, Baiyun District, Guangzhou, 510515 Guangdong China; 3 Division of Orthopaedics and Traumatology, Department of Orthopaedics, Guangdong Provincial Key Laboratory of Bone and Cartilage Regeneration Medicine, Nanfang Hospital, Southern Medical University, Guangzhou, 510515 China

**Keywords:** Musculoskeletal system, Medical imaging

## Abstract

The angle and position of the scapular glenoid are important in shoulder mechanics, the interpretation of diseases, and planning shoulder replacement surgery. In total shoulder replacement, understanding the bony parameters of the glenoid is also of considerable guiding significance for designing implant size and improving material adaptability. To compare glenoid parameters measured from skeletal scapula specimens with those measured by 3D modeling of CT scanning images, analyze correlations between these data, and draw conclusions to guide clinical treatment of shoulder joint injury and total shoulder joint replacement. The data of manual and CT measurements from the same Chinese dry glenoid was compared. Three-dimensional measurement data were collected from the Japanese population and compared with the Chinese population data generated in this study. There were no significant differences between manual measurement and CT measurement in the inclination angle, glenopolar angle, anteroposterior transverse diameter, upper to lower vertical diameter, and depth of the glenoid (P = 0.288, 0.524, 0.111, 0.194, and 0.055, respectively). Further, there were no significant differences between Japanese and Chinese glenoid bones in the upper and lower vertical diameters or anteroposterior transverse diameters (P > 0.05). There were no significant differences between CT and manual measurements, suggesting that the CT method may provide measurements very close to the actual specimen size. This result, however, indicated that the measurer should be careful when measuring the depth of the glenoid.

## Introduction

The angle and position of the scapular glenoid are important in shoulder mechanics and for the interpretation of diseases, such as glenohumeral instability and rotator cuff tear, as well as for planning shoulder replacement surgery^1,3,17^. In total shoulder replacement, understanding the bony parameters of the glenoid is also of considerable guiding significance for designing implant size and improving material adaptability^21^. Therefore, accurate preoperative measurement of the angle, glenoid position, and analysis of the postoperative recovery degree is crucial for pathological evaluation and successful total shoulder replacement^4,12,13^. In addition, the degree of glenopolar angle recovery can be an effective indicator for evaluating the prognosis for surgical success. Changes in the length (distance from the upper to the lower edge of the glenoid pelvis), diameter (distance from the leading edge to the posterior edge), and the depth of the glenoid pelvis are strongly related to glenoid joint instability^10^.

Due to the difference in body size between Asian and European people, implants currently used for total shoulder arthroplasty may not be suitable for populations with smaller anatomical bone structures^14^. Hence there is a need to collect and quantify anatomical data from Asian ethnicity populations (e.g., Chinese Han). At present, clinicians generally use a computed tomography (CT) and X-ray film images to complete such measurements.

Here, the difference between CT measurements and those from actual bone specimens was explored. The purpose of this study was to compare and analyze correlations between data collected using these two measurement methods.

## Materials and methods

### Materials

Sixty adult dried scapula specimens were provided by National Key Discipline of Human Anatomy, Southern Medical University. All body donors had provided written consent for collecting and using their specimens for medical research or teaching. All experimental methods and protocols performed in this study were in accordance with the relevant guidelines and regulations (Declaration of Helsinki) and approved by the School of Basic Medical Sciences, Southern Medical University. All specimens were complete in structure, without pathological changes or malformation that could affect data measurements. All the specimens were healthy glenoids, and if there were any obvious fractures or arthritic changes in the glenoids, they were excluded. Of specimens, 30 were left and 30 right scapulae.

### Measurements of glenoid from dry bone specimens

**Figure 1:**
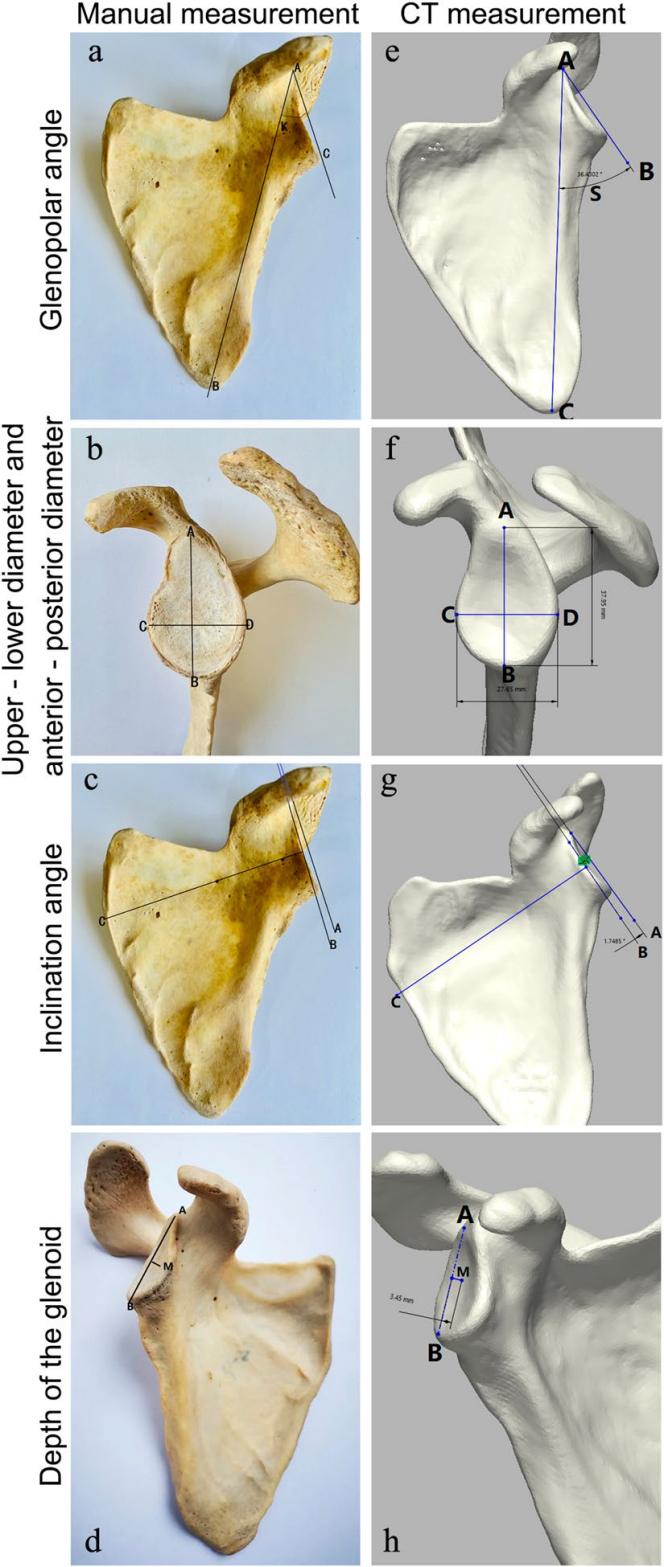
Measurements of the glenoid from dry bone specimens and CT images. (**a**,**e**) manual measurement and CT measurement of the glenopolar angle. Point A, upper edge of the glenoid. Point B, lower angle of the scapula. Point C, lower edge of the glenoid. Angle K, glenopolar angle (GPA). (**b**,**f**) Manual and CT measurement of the upper to lower vertical diameter and anteroposterior transverse diameter of the glenoid. AB, upper to lower diameter of glenoid; CD, anterior to posterior diameter of glenoid. (**c**,**g**) Manual and CT measurement of the inclination angle of glenoid. Line A, distance between the upper and lower margins of the glenoid. Line B, line passing through the midpoint of the glenoid; Line C is perpendicular to Line B. The angle between A and B was the inclination angle of glenoid. (**d**,**h**) Manual and CT measurements of glenoid depth. Line M, vertical line from the midpoint of the glenoid. AB, line from the upper to the lower margin of the glenoid. The distance from the end of Line M to Line AB from the midpoint of the glenoid socket is the glenoid depth.

Point A was defined as the upper edge of the glenoid, point B as the lower edge of the scapula, point C as the lowest point of the glenoid edge, and point D as the posterior edge of the glenoid. Angle K was the glenopolar angle between AB and AC (Fig. 1a), AB was the upper to lower diameter of the glenoid, and CD was anterior to the posterior diameter of the glenoid (Fig. 1b).

Line A was defined as the distance between the upper and lower margins of the glenoid, line B was the line passing through the midpoint of the glenoid pelvis, and line C was perpendicular to line B. Angle AC was the inclination angle of the glenoid (Fig. 1c).

Line M was defined as the vertical line from the midpoint of the glenoid, and line AB was the line from the upper to lower margin of the glenoid. The glenoid depth was the distance from one end of line M to line AB from the midpoint of the glenoid socket (Fig. 1d).

All data were measured three times by the same person. In addition, one surveyor measured the measurement of a parameter, which another senior researcher rechecked.

### Data acquisition, reconstruction, and measurement of the 3D model

Scapulae were scanned with CT using a 0.8-mm slice thickness. The data sets of Dicom images of the scapulae were imported into Mimics 21.0. and 3D models of the scapulae were created. The same parameters are measured in Fig. 1a–d directly on the software (Fig. 1e,h).

### Comparison with Japanese population data

Three-dimensional measurement data (upper to lower vertical diameter and anteroposterior transverse diameter of the glenoid) were collected from the Japanese population^14^ and compared with the Chinese population CT data generated in this study with GraphPad Prism 8.0 software (La Jolla, CA, USA).

### Statistical analysis

GraphPad Prism 8.0 software (La Jolla, CA, USA) was used for statistical data analysis. Normally distributed measurement data are presented as mean ± standard deviation (mean ± SD). *P* < 0.05 was considered statistically significant.

## Results

### Comparison of the results of manual and CT measurements

**Figure 2:**
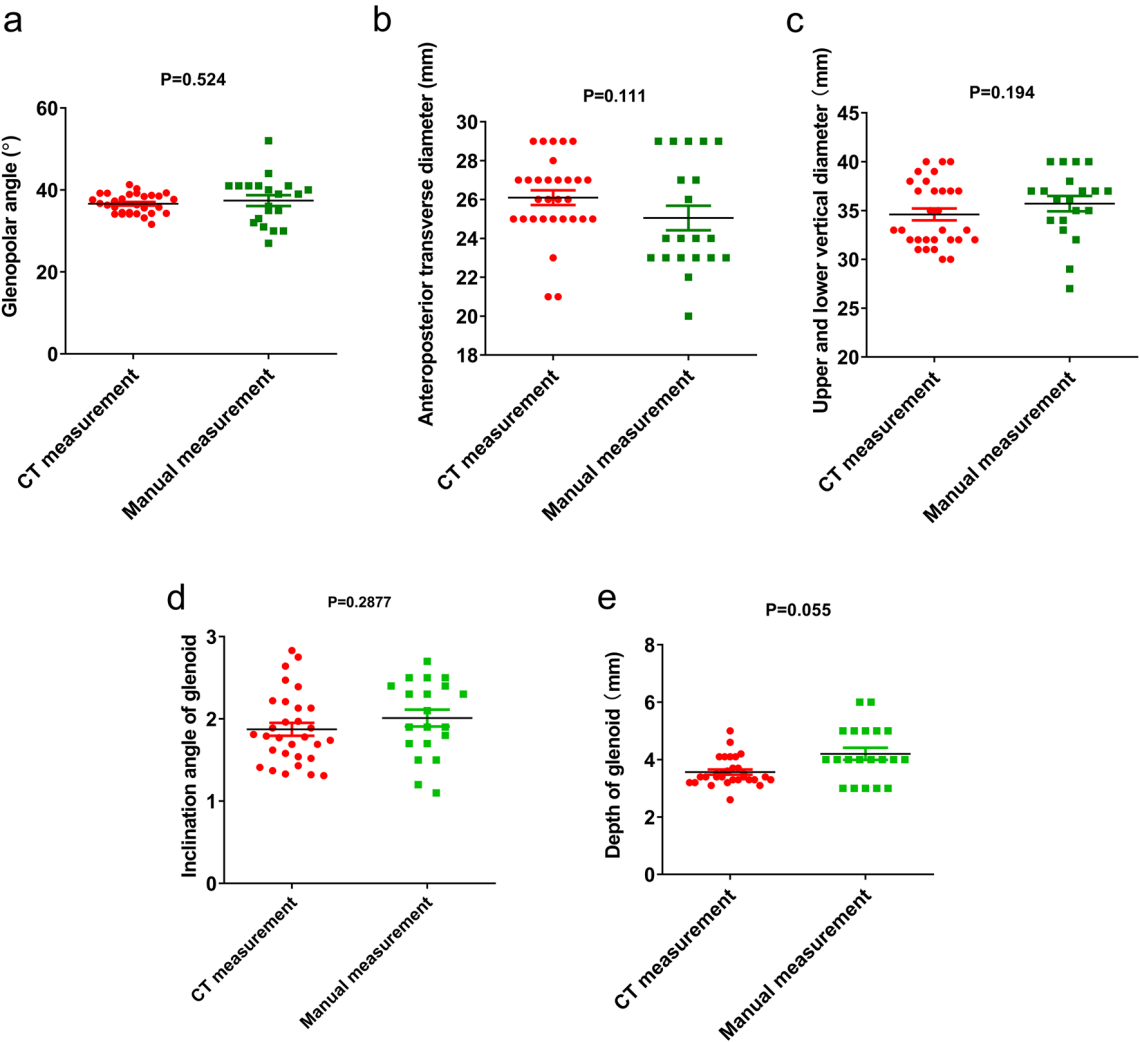
Comparisons of manual and CT measurements of the glenoid. (**a**) Glenopolar angle. (**b**) Anteroposterior transverse diameter of glenoid. (**c**) Upper to lower vertical diameter of glenoid. (**d**) Inclination angle of glenoid. (**e**) Depth of glenoid.

Comparisons of data from manual and CT measurements are presented in Fig. 2. The mean ± SD inclination angle of the glenoid determined by manual measurement was 2.01° ± 0.461°, while that assessed from CT imaging was 1.87° ± 0.43°; there was no significant difference between the two measurements (*P* = 0.288). Further, there was no significant difference in the median (range) glenopolar angle measured manually and from CT images, at 39° (32.25°–41°) and 36.55° (34.3°–38.75°), respectively (*P* = 0.524). The median (range) manual measurements of anteroposterior transverse diameter, upper to lower vertical diameter, and depth of glenoid were 24 (23–28.5) mm, 36.5 (34–37.75) mm, and 4.0 (3.5–5.0) mm, respectively, while the corresponding CT measurements were 26 (25–27) mm (*P* = 0.111), 33 (32–37.25) mm (P = 0.194), and 3.4 (3.3–3.7) mm (*P* = 0.055), respectively.

### Comparison of Japanese and Chinese population glenoid CT measurements

There were no significant differences in the glenoid's upper to lower vertical diameter or transverse diameter between the Chinese and Japanese population data (*P* > 0.05; Table 1).

**Table 1:** Comparison of glenoid parameters in individuals from Chinese and Japanese population CT data.

Diameter measurement (mm)	Chinese	Japanese	P-value
Anteroposterior transverse diameter (APTD)	26.10 ± 2.07	27.13 ± 1.70	0.023
Upper and lower vertical diameters (ULVD)	34.60 ± 3.31	34.47 ± 4.41	0.582

## Discussion

A precise understanding of the anatomy of the glenoid cavity is necessary for treating shoulder instability, recurrent dislocation of the glenoid cavity, osteogenic defect of the glenoid cavity, artificial shoulder replacement, and other conditions^7,11^. Inappropriate implants in reverse shoulder arthroplasty can easily lead to surgical failure or postoperative discomfort. Comprehensive, accurate, and reproducible measurement methods and indicators are needed to evaluate shoulder joint and glenoid morphology abnormalities. Also, these methods are helpful to guide clinical decisions and help predict shoulder joint diseases, select appropriate treatment methods, and evaluate patient prognosis. Radiographic evaluation and measurement indices can comprehensively reflect the shoulder joint cavity. Plain X-ray film, magnetic resonance imaging, and CT 3D reconstruction are used to measure the glenoid^2,8,20^, and various measurement methods and definition lines are applied. The measurements using these approaches were compared with those directly assessing the bone structure. Therefore, this study was to compare and analyze data collected using two measurement methods.

The osseous parameters measured in this study included the inclination angle of the glenoid, which refers to the angle between a vertical line from the medial edge of the scapula and the line of the upper and lower articular glenoid and expresses the relationship of glenoid inclination to the coronal plane of the scapula. The glenopolar angle is formed by the connection between the upper and lower end of the glenoid and the connection between the upper end of the glenoid and the subscapular angle. The normal range of the glenopolar angle is 30°–45°; glenopolar angle < 20° and inclination angle of glenoid > 20°, indicating serious deformity of the rotation of the glenoid requiring surgery^9,22^. Previously, Daggett et al.,^6^ compared inclination angle measurements of glenoids generated from radiographs with those from CT and concluded that CT measurements were more accurate. The results indicated no significant difference between CT and manual measurements, suggesting that CT may generate measurements very close to the actual specimen size. The results suggest that although there is no significant difference in the depth of glenoid between measurements made using CT and those from actual bone specimens (*P* = 0.055), However, this result indicated that the measurer should be careful in measuring the depth of glenoid to determine the size of artificial shoulder replacement and the length of fastening screw and fixed depth.

Rosales-Rosales et al.,^18^ measured glenoid size in a normal Hispanic population compared to other populations, as well as conducting a size-fit study of implants for shoulder replacement, and concluded that the glenoid was significantly larger in males, who were similar in size (height: 28.78 ± 2.93 mm; width: 20.27 ± 2.46 mm) to the Caucasian population (average height: 27.87 ± 5.5 mm; width: 29.35 ± 5.2 mm)^16^, but smaller than the U.S. population (height: 33.9 ± 3.9 mm; width: 28.6 ± 3.8 mm)^15^; it was anticipated that the data presented may help improve shoulder prosthesis design for the southern Spanish population. In this study, we collected data of anteroposterior transverse diameter and upper to lower vertical diameter, which most directly reflects glenoid size, from a Japanese population and compared them with the data from Chinese individuals. The data in this study are consistent with previous studies showing that the glenoid size does not differ significantly between Chinese and Japanese people^18,19^.

Matsuki et al.,^14^ measured the glenoid of healthy volunteers aged 20–40 years and subjects over 50 years old with shoulder disease in a Japanese population. Although significant osteoarthritis was excluded, it was noted during measurement that some patients had minor osteophytes or hypertrophy of glenoid margin, possibly due to aging or pathological changes. These morphological changes may have had some influence on measurement^14^. Further, glenoid size is reported to be larger in osteoarthritic shoulders^14,16,21^. Cabezas et al.,^5^ studied morphological differences between North Americans and East Asians using 3D CT reconstruction, and observed that glenoid morphology measurements were lower in the Asian population than those in North Americans. In addition, some publications have compared morphometric data from Thai or Chinese populations with those from North Americans, and observed that the morphometric measurement data from North American populations were larger than those from Asian populations^1,5^. These ethnic morphological differences suggest the potential for differences in the glenoid of the shoulder joint.

The limitations of this study include the sample size and the inability to distinguish the side, gender, and age of analyzed subjects.

## Conclusions

There is no significant difference between direct manual and CT measurements of the glenoid; however, this result indicated that the measurer should be careful in measuring the depth of the glenoid.

## Data Availability

The datasets used and/or analyzed during the current study available from the corresponding author on reasonable request.

## References

[1] Aroonjarattham P, Jiamwatthanachai P, Mahaisavariya B, Kiatiwat T, Aroonjaratthammd K, Sitthiseripratip K (2009). Three-dimensional morphometric study of the Thai proximal humerus: Cadaveric study. J. Med. Assoc. Thai..

[2] Bodrogi A, Athwal GS, Howard L, Zhang T, Lapner P (2019). A reliable method of determining glenohumeral offset in anatomic total shoulder arthroplasty. J. Shoulder Elbow Surg..

[3] Boileau P, Cheval D, Gauci M, Holzer N, Chaoui J, Walch G (2018). Automated three-dimensional measurement of glenoid version and inclination in arthritic shoulders. J. Bone Joint Surg..

[4] Boileau P, Gauci M, Wagner ER, Clowez G, Chaoui J, Chelli M, Walch G (2019). The reverse shoulder arthroplasty angle: A new measurement of glenoid inclination for reverse shoulder arthroplasty. J. Shoulder Elbow Surg..

[5] Cabezas AF, Krebes K, Hussey MM, Santoni BG, Kim HS, Frankle MA, Oh JH (2016). Morphologic variability of the shoulder between the populations of North American and East Asian. Clin. Orthop. Surg..

[6] Daggett M, Werner B, Gauci MO, Chaoui J, Walch G (2016). Comparison of glenoid inclination angle using different clinical imaging modalities. J. Shoulder Elbow Surg..

[7] Denard PJ, Raiss P, Sowa B, Walch G (2013). Mid- to long-term follow-up of total shoulder arthroplasty using a keeled glenoid in young adults with primary glenohumeral arthritis. J. Shoulder Elbow Surg..

[8] Goldberg SS, Baranek ES, Korbel KC, Blaine TA, Levine WN (2021). Anatomic total shoulder arthroplasty using a stem-free ellipsoid humeral implant in patients of all ages. J. Shoulder Elbow Surg..

[9] Hess F, Zettl R, Smolen D, Knoth C (2019). Decision-making for complex scapula and ipsilateral clavicle fractures: A review. Eur. J. Trauma Emerg..

[10] Hong J, Huang Y, Ma C, Qu G, Meng J, Wu H, Shi M, Wang Y, Zhou C, Chen Z, Yan S, Wang W (2019). Risk factors for anterior shoulder instability: A matched case-control study. J. Shoulder Elbow Surg..

[11] Iannotti JP, Ricchetti ET, Rodriguez EJ, Bryan JA (2013). Development and validation of a new method of 3-dimensional assessment of glenoid and humeral component position after total shoulder arthroplasty. J. Shoulder Elbow Surg..

[12] Kwon YW, Powell KA, Yum JK, Brems JJ, Iannotti JP (2005). Use of three-dimensional computed tomography for the analysis of the glenoid anatomy. J. Shoulder Elbow Surg..

[13] Lenart BA, Freedman R, Van Thiel GS, Dhawan A, McGill KC, Basu S, Meyer JR, Provencher CMT, Cole BJ, Romeo AA, Verma NN (2014). Magnetic resonance imaging evaluation of normal glenoid length and width: An anatomic study. Arthrosc. J. Arthrosc. Relat. Surg..

[14] Matsuki K, Sugaya H, Hoshika S, Ueda Y, Takahashi N, Tokai M, Banks SA (2019). Three-dimensional measurement of glenoid dimensions and orientations. J Orthop. Sci..

[15] McPherson EJ, Friedman RJ, An YH, Chokesi R, Dooley RL (1997). Anthropometric study of normal glenohumeral relationships. J Shoulder Elbow Surg.

[16] Moineau G, Levigne C, Boileau P, Young A, Walch G (2012). Three-dimensional measurement method of arthritic glenoid cavity morphology: Feasibility and reproducibility. Orthopaed. Traumatol. Surg. Res..

[17] Mook WR, Petri M, Greenspoon JA, Horan MP, Dornan GJ, Millett PJ (2016). Clinical and anatomic predictors of outcomes after the latarjet procedure for the treatment of anterior glenohumeral instability with combined glenoid and humeral bone defects. Am. J. Sports Med..

[18] Rosales-Rosales L, Rosales-Varo AP, García-Espona MA, Roda-Murillo O, Montesinos I, Hernandez-Cortés P (2019). Estudio antropométrico de la glena humana en una población española normal. Revista Española de Cirugía Ortopédica y Traumatología.

[19] Shi L, Griffith JF, Huang J, Wang D (2013). Excellent side-to-side symmetry in glenoid size and shape. Skelet. Radiol..

[20] Strauss EJ, Roche C, Flurin P, Wright T, Zuckerman JD (2009). The glenoid in shoulder arthroplasty. J. Shoulder ELB Surg..

[21] Walch G, Mesiha M, Boileau P, Edwards TB, Levigne C, Moineau G, Young A (2013). Three-dimensional assessment of the dimensions of the osteoarthritic glenoid. Bone Joint J..

[22] Yadav V, Khare GN, Singh S, Kumaraswamy V, Sharma N, Rai AK, Ramaswamy AG, Sharma H (2013). A prospective study comparing conservative with operative treatment in patients with a 'floating shoulder' including assessment of the prognostic value of the glenopolar angle. Bone Joint J.

