# Author Correction: Anatomic measurement of osseous parameters of the glenoid

**DOI:** 10.1038/s41598-022-21565-x

**Published:** 2022-10-03

**Authors:** Jing Zhou, Bin Zhong, Rongmei Qu, Lei Qian, Zeyu Li, Chang Liu, Zhaoming Xiao, Guangwei Xu, Haibin Liang, Kuanhai Wei, Jun Ouyang, Jingxing Dai

**Affiliations:** 1 Department of Anatomy, Youjiang Medical University for Nationalities, Baise, 533000 China; 2 Guangdong Provincial Key Laboratory of Medical Biomechanics and Guangdong Engineering Research Center for Translation of Medical 3D Printing Application and National Virtual and Reality Experimental Education Center for Medical Morphology (Southern Medical University) and National Key Discipline of Human Anatomy, School of Basic Medical Sciences, Southern Medical University, 1023 Shatai South Road, Baiyun District, Guangzhou, 510515 Guangdong China; 3 Division of Orthopaedics and Traumatology, Department of Orthopaedics, Guangdong Provincial Key Laboratory of Bone and Cartilage Regeneration Medicine, Nanfang Hospital, Southern Medical University, Guangzhou, 510515 China

Correction to: *Scientific Reports* https://doi.org/10.1038/s41598-022-17783-y, published online 04 August 2022

The original version of this Article contained an error in Affiliation 1, which was incorrectly given as ‘Department of Anatomy, Youjiang Medical University for Nationalities, Baishe, 533000, China.’

The correct affiliation is listed below:

Department of Anatomy, Youjiang Medical University for Nationalities, Baise, 533000, China.

The original Article has been corrected.

